# Comparison of Different Machine Learning Techniques to Predict Diabetic Kidney Disease

**DOI:** 10.1155/2022/7378307

**Published:** 2022-04-01

**Authors:** Satish Kumar David, Mohamed Rafiullah, Khalid Siddiqui

**Affiliations:** Strategic Center for Diabetes Research, College of Medicine, King Saud University, Riyadh, Saudi Arabia

## Abstract

**Background:**

Diabetic kidney disease (DKD), one of the complications of diabetes in patients, leads to progressive loss of kidney function. Timely intervention is known to improve outcomes. Therefore, screening patients to identify high-risk populations is important. Machine learning classification techniques can be applied to patient datasets to identify high-risk patients by building a predictive model.

**Objective:**

This study aims to identify a suitable classification technique for predicting DKD by applying different classification techniques to a DKD dataset and comparing their performance using WEKA machine learning software.

**Methods:**

The performance of nine different classification techniques was analyzed on a DKD dataset with 410 instances and 18 attributes. Data preprocessing was carried out using the PartitionMembershipFilter. A 10-fold cross validation was performed on the dataset. The performance was assessed on the basis of the execution time, accuracy, correctly and incorrectly classified instances, kappa statistics (K), mean absolute error, root mean squared error, and true values of the confusion matrix.

**Results:**

With an accuracy of 93.6585% and a higher K value (0.8731), IBK and random tree classification techniques were found to be the best performing techniques. Moreover, they also exhibited the lowest root mean squared error rate (0.2496). There were 15 false-positive instances and 11 false-negative instances with these prediction models.

**Conclusions:**

This study identified IBK and random tree classification techniques as the best performing classifiers and accurate prediction methods for DKD.

## 1. Introduction

Advancements in information technology have led to the creation of enormous volumes of data. Besides, the developments in healthcare database management systems have resulted in a vast number of medical databases. Managing large volumes of heterogeneous data and creating useful knowledge from them has become an important field of research known as data mining. It is a way of discovering innovative, valuable, valid, and reasonable patterns in data [1]. There are two data mining techniques, namely, unsupervised and supervised learning techniques. Unsupervised learning techniques identify novel patterns with minimum human supervision. It works with unlabeled data and looks for a hidden pattern in the data. It builds a model based on the results obtained. A commonly used unsupervised technique is clustering [2]. Supervised learning techniques require labeled training data. It analyzes the training example data to deduce a pattern that can be applied to new example data. Classification, statistical regression, and association rules are commonly used supervised learning techniques in medical and clinical research [3]. Classification methods are used to classify, detect, and analyze disease datasets to build a prediction model [4].

Machine learning is an integral part of artificial intelligence (AI) that allows the systems to perform a specific task without using explicit programming. It works by creating patterns and inferences by building a model based on a training dataset. Machine learning involves developing computer programs that can use data to learn for themselves [5]. Waikato environment for knowledge analysis (WEKA) is a data mining software that contains algorithms for data analysis and predictive modeling. It consists of all the major learning techniques for classification and regression, such as Bayesian classifiers, decision trees, rule sets, support vector machines, logistic and multilayer perceptrons, linear regression, and nearest-neighbor methods. It also has “meta-learners” such as bagging, stacking, boosting, and schemes that perform automatic parameter tuning using cross-validation, cost-sensitive classification, etc. [6]. A comparison of the advantages and disadvantages of these classifiers in presented in Supplementary table 1.

Learning algorithms need to be validated as the dataset may not be truly representing the population. Cross-validation hold-out set or resubstitution are some of the validation techniques. There are standard quantitative performance parameters such as accuracy and root mean squared error available in WEKA software. It also provides graphical performance indicators such as receiver operating characteristic curves and precision-recall curves. The visualization tools available in WEKA allow the identification of outliers [7].

Diabetic kidney disease (DKD) is one of the most common complications of diabetes that causes increased mortality and morbidity in patients [8]. It occurs in 20–40% of people with diabetes. DKD is the single largest cause of end-stage renal disease (ESRD) worldwide and has become an enormous burden on healthcare systems [9]. Patients in the early stage of diabetic nephropathy are characterized as microalbuminuria (albumin-to-creatinine ratio (ACR) of 30–299 mg/g). In many patients, it usually progresses to macroalbuminuria (albumin-to-creatinine ratio (ACR) of ≥300 mg/g) followed by ESRD. However, screening patients early for diabetic nephropathy will help delay the onset of microalbuminuria and may prevent the progression of micro to macroalbuminuria and ESRD [10]. Standard methods to detect renal impairment involve specialized blood and urine tests. However, data mining techniques can be applied to the available datasets to establish a prediction model that can be used for detecting DKD cases.

AI technique was used to build a predictive model that detected DKD aggravation with 71% accuracy [11]. Machine learning methods were used to predict the initiation of renal replacement therapy in chronic kidney disease patients. Only the comorbidity data were used to build the prediction model. The area under the receiver operating characteristic curve for predicting the initiation of renal replacement therapy within a year from CKD diagnosis was found to be 0.773 [12]. An AI-based recursive rule extraction technique was used to derive lower urinary albumin to creatinine ratio cut-offs for the early detection of DKD. This technique identified two cutoff values with an accuracy of 77.56% [13]. Ravizza et al. developed a model from real-world data of people with type 2 diabetes for detecting chronic kidney disease. The area under the receiver operating characteristic curve of the model was 0.7937 [14].

## 2. Recent Works

Early detection of diabetic retinopathy was developed using deep learning techniques. The dataset was preprocessed before the classification. A standard scalar technique was used to normalize the date, and principal component analysis was used to extract the data. Dimensionality reduction was carried out using the firefly algorithm. The accuracy of the deep neural network model was found to be 97% and it outperformed other classification techniques such as support vector machines, KNN, decision tree, NB, and XGBoost-based models [15]. Chowdhury et al. analyzed the data from the epidemiology of diabetes interventions and complications clinical trials to develop a prediction model based on different machine learning algorithms. It included 1375 patients with type 1 diabetes and 19 attributes. The random forest model was found to be best (96%), followed by a light gradient-boosted machine (95%) [16]. XGBoost and random forest algorithms were used to develop a model to predict the 5-year risk of CKD. The dataset included 88,973 individuals. The AUC was 0.75 for predicting any stage of CKD and 0.82 for severe endpoints. The models outperformed the Centers for Disease Control and Prevention (CDC) risk score [17].

The currently available techniques use specific methods for building the DKD prediction models. A comparative analysis is needed to identify an accurate method for the prediction of DKD. In this study, we aimed to identify an accurate classification technique for predicting DKD by comparing different classification techniques applied to a DKD dataset using WEKA machine learning software. Here we report the use of a machine learning technique to detect patients with DKD using known cases of DKD as a training dataset.

## 3. Materials and Methods

**Figure 1:**
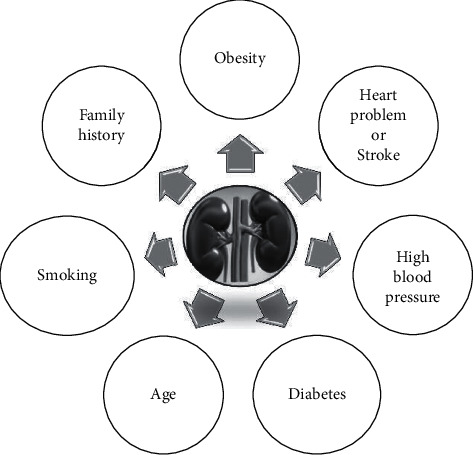
Risk factors affecting diabetic kidney disease.

Clinical and biochemical data of patients who had DKD were gathered for this study.  Figure 1 shows the risk factors affecting diabetic kidney disease.

**Figure 2:**
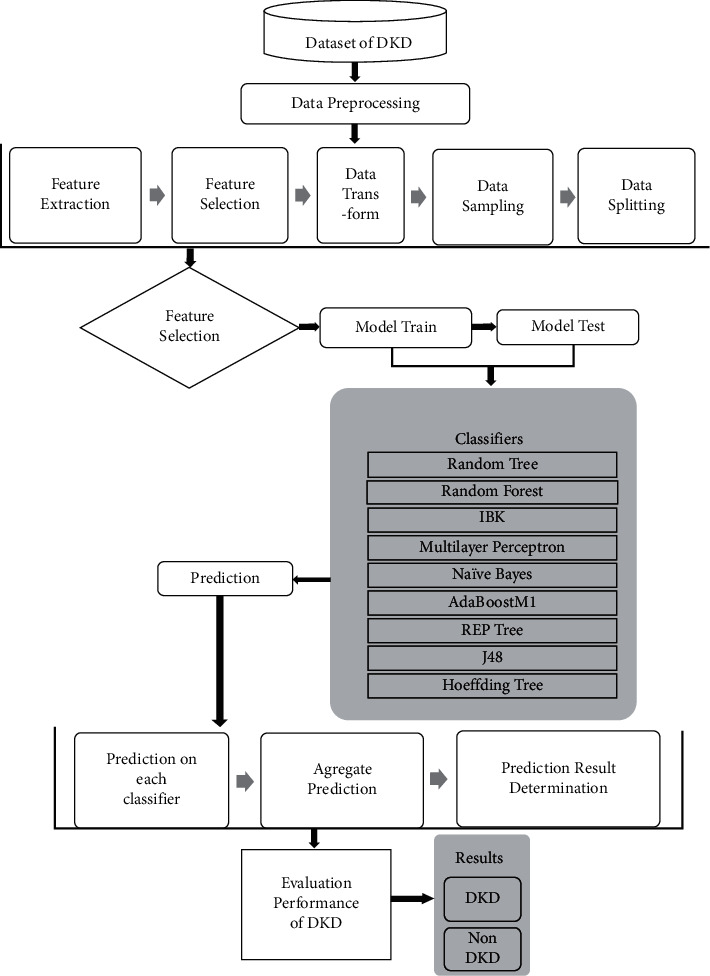
Block diagram of the proposed research.

**Figure 3:**
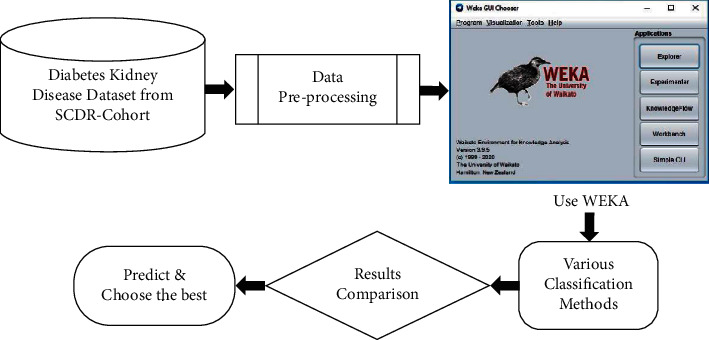
Schematic illustration of the methodology used for identifying the best performing classification technique.

The data collected were transformed to data types ARFF file. ARFF is an acronym that stands for attribute-relation file format. It is an extension of the CSV file format where a header is used. This header provides metadata about the data types in the columns. The data was saved with an extension of CSV from Microsoft Excel and then opened in WEKA using the “ArffViewer” under the “Tools” option to save it with an ARFF extension. This conversion has to be done in order for the data to be used in WEKA. A 10-fold cross-validation was performed on the dataset, and then the data was analyzed using WEKA. Different machine learning classification techniques were applied, and the outcomes were compared (Figure 2). The best performing technique was identified based on findings to predict DKD (Figure 3).

### 3.1. Dataset

The diabetic kidney disease dataset was gathered from our previous DKD cohort [18]. There are 410 instances and 18 attributes (14 numeric and 4 nominal) that were used in the analysis of the prediction of DKD. The dataset attributes are age (years), gender (male/female), serum albumin (mg/dL), sodium (mmol/L), potassium (mmol/L), urea (mg/dL), glucose (mg/dL), creatinine (mg/dL), HbA1c (%) Hb (g/dL), white blood cell counts (WBCs) (10^9^/L), red blood cell counts (RBCs) (10^12^/L) Hb (%), platelets counts (10^9^/L) (M/µl), systolic BP sitting condition (mmHg), diastolic BP sitting condition (mmHg), hypertension (yes/no), and retinopathy (yes/no). The attribute nephropathy was classified into two classes as DKD and not DKD. 410 patients with diabetes were classified according to their urinary albumin excretion creatinine ratio (ACR) using American Diabetes Association (ADA) criteria for diabetic nephropathy stage cutoff and eGFR values.

### 3.2. Preprocessing

Preprocessing is a data mining technique that involves transforming raw data into an understandable format. WEKA now also has a PartitionMembershipFilter that can apply any PartitionGenerator to a given dataset to obtain these vectors for all instances. For preprocessing, a partition membership filter is used.

**Figure 4:**
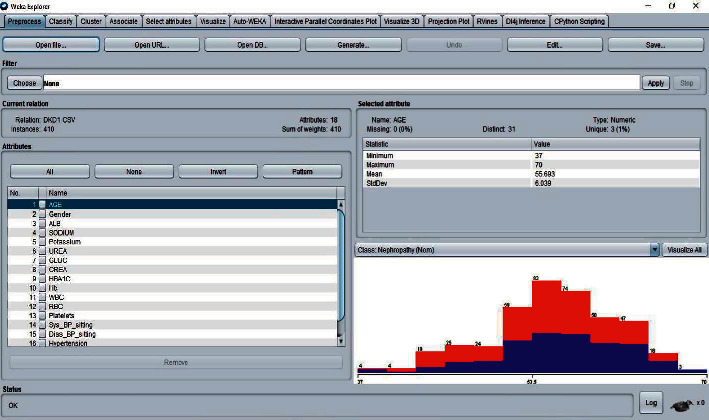
WEKA-Explorer window.

There are four interfaces to WEKA which can be started from the main GUI Chooser window.  Figure 4 shows the DKD dataset after loading in the explorer window of the WEKA tool. The visualization section with blue and red code indicates the data in the form of a graph. In WEKA, results are partitioned into several subitems for easier analysis, evaluation, and simulation. It begins with partitioning correctly and incorrectly classified instances in numeric and percentage values, followed by the computation of Kappa statistics, mean absolute error, and root mean squared error in numeric values.

### 3.3. Classification

Classification is a data mining algorithm to find out the output of a new data instance. In this study, different classifiers were applied on the DKD dataset for comparing their accuracy, correctly classified instances, incorrectly classified instances, error rate, and execution time to evaluate overall performance and identify the best classifier for DKD prediction. The nine different classification techniques that were used in the study are as follows: random forest, J48, Naïve Bayes, REP tree, random tree, multilayer perceptron, AdaBoostM1, Hoeffding Tree, and IBK.

**Figure 5:**
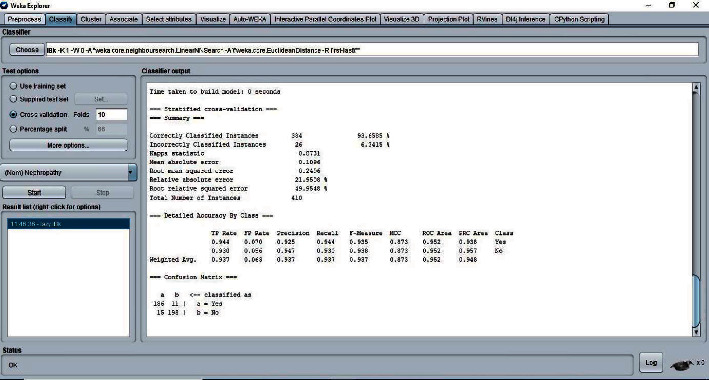
Classifier IBK result.

**Figure 6:**
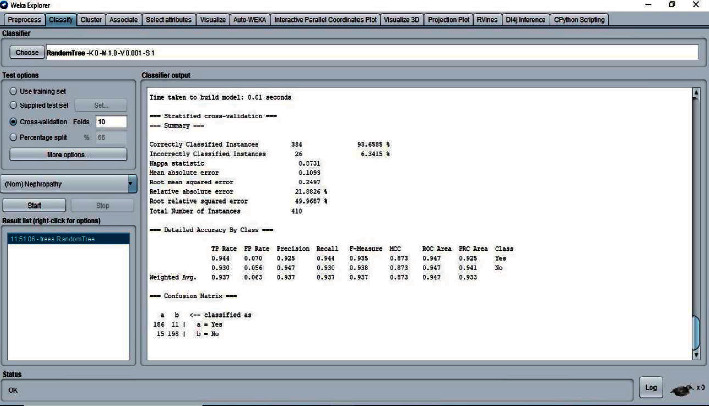
Classifier random tree result.

**Figure 7:**
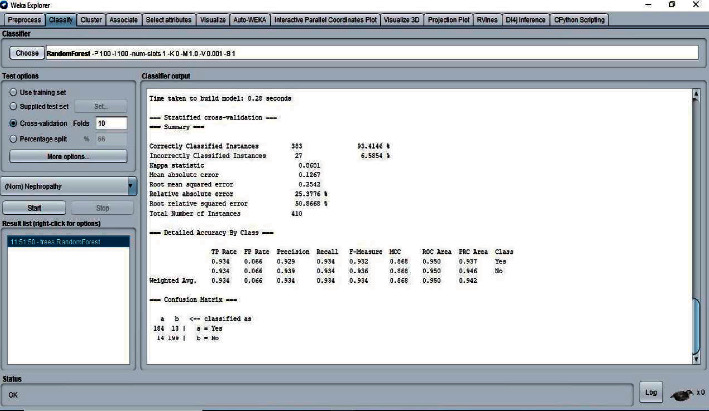
Classifier random forest result.

**Figure 8:**
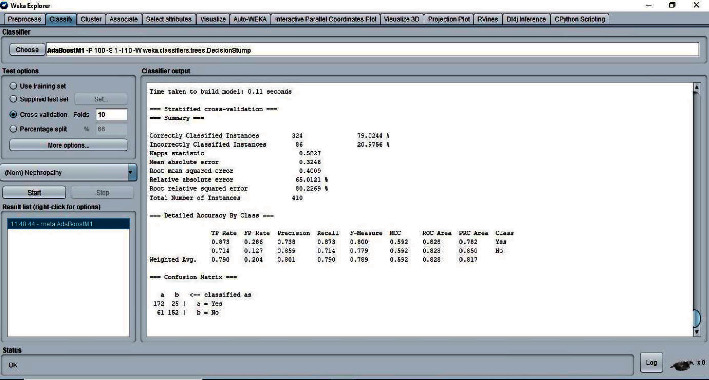
Classifier AdaBoostM1 result.

The 10-fold cross-validation is the standard method of evaluation for different machine learning techniques. The dataset was divided into ten equal subsets, with one subset used for testing and one for training. This was continued until all the subsets had been used for testing. We applied the 10-fold cross-validation test for evaluating the performance of different classifiers, as shown in Figures 5–8. The predictions for each test instance are then listed in the “Classifier Output” pane in WEKA.

WEKA machine learning software was used for learning different models, preprocessing, and feature selection schemes to identify the best classification method by comparison.

## 4. Results and Discussion

Table 1 shows the comparative results from the10-fold cross-validation testing of different classifiers.

Results show that IBK and multilayer perceptron are the fastest and slowest classifiers, respectively. The accuracy of the classifiers is comparable to each other. However, the IBK and random tree methods are the most accurate (93.6585%). The number of correctly classified instances in the IBK method is the highest, followed by the random tree and random forest methods. In the case of incorrectly classified instances, the IBK and random tree methods have the lowest instances. AdaBoostM1 was found to be the lowest in accuracy and correctly classified instances and has the highest incorrectly classified instances among all the classifiers. Both IBK and random tree techniques are found to be superior to other classifiers in terms of execution time, accuracy, correctly classified instances, and incorrectly classified instances.

Table 2 shows the results of Kappa statistics (K), mean absolute error (MAE), and root mean squared error (RMSE) for the different classification methods.

A Kappa statistics (K) value greater than 0 means the classifier is doing better than the chance of agreement. IBK and random tree have shown greater K values than the other classifiers in this study. Mean absolute error (MAE) values indicate how close the prediction result is to the actual values. The results show that the random tree classifier has the lowest MAE. Therefore, the prediction result of the random tree classifier is very close to the true cases of DKD. Root mean squared error (RMSE) rates are used to identify the best classification technique when their MAE values are found to be similar. The IBK classifier achieved the lowest RMSE rate when compared to other classifiers. With the lower K value and higher MAE and RMSE rates, the prediction values of AdaBoostM1 are considered to be the least significant. On the other hand, both the IBK and random tree techniques are found to achieve better prediction results, and the other classifiers' prediction results are average.

Table 3 shows the confusion matrix of the classification methods.

The confusion matrix table describes the performance of different classification models on the DKD test dataset for which the actual DKD cases are known. The IBK classifier correctly identified 93.0% of patients as not having DKD and 94.42% of patients as having DKD. There were 7.46% of false-positive cases and 5.26% of false-negative cases. It has the best prediction performance among all the classifiers investigated. Our results are comparable to the previously reported prediction models for DKD (Table 4). A maximum accuracy level was achieved when a recursive feature elimination technique was used to choose the attributes [19].

Many studies have reported different classifiers for the prediction of DKD. A probabilistic neural network method was found to provide better classification and prediction performance in determining the stages of DKD [23]. BayesNet and REP tree algorithms showed accurate performance in the prediction of chronic kidney disease [24]. However, in another study, J48 was found to be suitable for screening DKD [20]. The gradient boosting classifier was the accurate method in the detection of DKD with the least number of predictors [25]. C4.5 classifier efficiently predicted chronic kidney disease from a high-dimensional dataset [26]. A review found that many researchers have used KNN, ANN, Naïve Bays, SVM, and decision tree (J48, C4.5) for a prediction of chronic kidney disease from the given dataset. The highly accurate classifier was SVM (98.5%), and the least accurate was the Bayes network (57.5%) [27].

The AdaBoost classifier algorithm was found to be highly accurate (0.917) for the prediction of diabetic nephropathy in a dataset of 884 patients and 70 attributes. When the attributes were decreased to the top 5 only, the performance was not affected [28]. Our results show that IBK and random tree classifiers with a dataset of 410 patients and 18 attributes achieved an accuracy of 93.6585%. A systematic review on machine learning methods for prediction of diabetes complications found that random forest algorithm is the overall best prediction performing classifier [29]. We found that the IBK algorithm is the best prediction performing classifier, in general, IBK means KNN algorithm is one of the best classifiers.

Random forest and simple logistic regression methods were shown to have better performance in the prediction of nephropathy in type 2 diabetes from the ACCORD trial dataset [30]. Pasadana et al. also found the random forest classifier to be the best technique for DKD prediction [31]. Random forest regression was used to build a model with data from real-world electronic medical records to predict future kidney functions accurately and provide clinical decision support [32]. In the present study, based on the performance evaluation of classifiers on the DKD dataset, we found that the IBK and random tree classifiers exhibited the best performance compared to the other classifiers like J48, Naïve Bayes, REP tree, AdaBoostM1, Hoeffding Tree, random forest, and multilayer perceptron.

The predictive models can be used in real-life situations when extensive invasive tests are not possible. High-risk patients may be identified using the available dataset. Our predictive model was developed using easily available routine laboratory parameters. Therefore, screening patients to identify those who are vulnerable for developing kidney disease is possible in primary clinics. It will help the clinicians to decide on starting intensive preventive therapy for the high-risk patients.

## 5. Conclusions

In this paper, we have applied different classification techniques to a DKD dataset for the prediction of DKD. IBK and random tree classification techniques are identified as the best performing classifiers and accurate prediction methods for DKD. These techniques may be used to detect DKD patients with easily available basic lab parameters. Using data mining techniques for predictive analytics, especially in the medical field, can save time and money. Our study compared nine different types of classification algorithms using the WEKA data mining tool to identify the best classifier that is suitable for the DKD dataset. These models will be useful in the early prediction of chronic kidney disease to take proactive interventions and reduce the mortality and morbidity associated with the disease. The prediction models may be developed further for predicting the progression of DKD in vulnerable patients.

## Figures and Tables

**Table 1:** Comparison of different classifiers applied on the DKD dataset.

Classifier	Execution time (seconds)	Accuracy (%)	Correctly classified instances	Incorrectly classified instances
IBK	0	93.6585	384	26
Random tree	0.01	93.6585	384	26
Random forest	0.28	93.4146	383	27
Multilayer perceptron	8.3	93.1707	382	28
J48	0.13	89.7561	368	42
Hoeffding tree	0.04	86.0976	353	57
REP tree	0.08	85.122	349	61
Naïve bayes	0.01	80.9756	332	78
AdaBoostM1	0.11	79.0244	324	86

**Table 2:** Classification results from WEKA.

Classifier	Kappa statistics (K)	Mean absolute error (MAE)	Root mean squared error (RMSE)
IBK	0.8731	0.1096	0.2496
Random tree	0.8731	0.1093	0.2497
Random forest	0.8681	0.1267	0.2542
Multilayer perceptron	0.8633	0.1117	0.2513
J48	0.7947	0.1595	0.3074
Hoeffding tree	0.7223	0.1389	0.3696
REP tree	0.7025	0.2194	0.3565
Naïve bayes	0.6199	0.1899	0.4261
AdaBoostM1	0.5827	0.3246	0.4009

**Table 3:** Confusion matrix of different classifiers.

Classifiers	Prediction		Actual state (clinical definition) (197 DKD and 213 not DKD)
	DKD	Not DKD	
IBK	186	11	DKD
	15	198	NOT DKD
Random tree	186	11	DKD
	15	198	NOT DKD
Random forest	184	13	DKD
	14	199	NOT DKD
Multilayer perceptron	184	13	DKD
	15	198	NOT DKD
J48	174	23	DKD
	19	194	NOT DKD
Hoeffding tree	36	177	DKD
	81	116	NOT DKD
REP tree	171	26	DKD
	35	178	NOT DKD
Naïve bayes	165	32	DKD
	46	167	NOT DKD
AdaBoostM1	172	25	DKD
	61	152	NOT DKD

**Table 4:** Comparison of recent works of predictive models for diabetic kidney disease or diabetic nephropathy.

Source	Dataset	Model	Complication	Accuracy (%)
Sobrinho et al., 2020 [20]	114 instances and 8 attributes	J48 decision tree	DKD	95
Senan et al., 2021 [19]	400 instances and 24 attributes	Recursive feature elimination to choose attributes followed by random forest classification	DKD	100
Almansour et al., 2019 [21]	400 instances and 24 attributes	Artificial neural network	CKD	99.7
Khanam and foo, 2021 [22]	768 instances and 9 attributes	Neural network	Diabetes	88.6
Our study	410 instances and 18 attributes	IBK and random tree	DKD	93.6585

## Data Availability

The data are available from the corresponding author on reasonable request.

## Abbreviations

DKD: Diabetic kidney disease
K: Kappa statistics
MAE: Mean absolute error
RMSE: Root mean squared error
WEKA: Waikato environment for knowledge analysis
ESRD: End-stage renal disease
AI: Artificial intelligence.

## Supplementary Materials

Supplementary Materials Supplementary Table 1. Comparison of advantages and disadvantages of different classifiers used in our study.

## References

[1] Fayyad U., Stolorz P. (1997). Data mining and KDD: Promise and challenges. *Future Generation Computer Systems*.

[2] Guerra L., McGarry L. M., Robles V., Bielza C., Larrañaga P., Yuste R. (2011). Comparison between supervised and unsupervised classifications of neuronal cell types: a case study. *Developmental Neurobiology*.

[3] Yoo I., Alafaireet P., Marinov M. (2012). Data mining in healthcare and biomedicine: a survey of the literature. *Journal of Medical Systems*.

[4] Polat H., Danaei Mehr H., Cetin A. (2017). Diagnosis of chronic kidney disease based on support vector machine by feature selection methods. *Journal of Medical Systems*.

[5] Corporation O. (2018). *Machine Learning-Based Adaptive Intelligence: The Future of Cybersecurity Executive Summary. January*.

[6] David S. K., Saeb A., Rubeaan K. Al. (2013). Comparative analysis of data mining tools and classification techniques using WEKA in medical Bioinformatics. *Computer Engineering and Intelligent*.

[7] Bouckaert R. R., Frank E., Hall M. (2013). *WEKA Manual for Version 3*.

[8] Lee S.-Y., Choi M. E. (2015). Urinary biomarkers for early diabetic nephropathy: beyond albuminuria. *Pediatric Nephrology*.

[9] Couser W. G., Remuzzi G., Mendis S., Tonelli M. (2011). The contribution of chronic kidney disease to the global burden of major noncommunicable diseases. *Kidney International*.

[10] American Diabetes Association (2005). Standards of medical care in diabetes. *Diabetes Care*.

[11] Makino M., Yoshimoto R., Ono M. (2019). Artificial intelligence predicts the progression of diabetic kidney disease using big data machine learning. *Scientific Reports*.

[12] Dovgan E., Gradišek A., Luštrek M. (2020). Using machine learning models to predict the initiation of renal replacement therapy among chronic kidney disease patients. *PLoS One*.

[13] Hayashi Y. (2019). Detection of lower albuminuria levels and early development of diabetic kidney disease using an artificial intelligence-based rule extraction Approach. *Diagnostics*.

[14] Ravizza S., Huschto T., Adamov A. (2019). Predicting the early risk of chronic kidney disease in patients with diabetes using real-world data. *Nature Medicine*.

[15] Gadekallu T. R., Khare N., Bhattacharya S. (2020). Early detection of diabetic retinopathy using pca-firefly based deep learning model. *Electronics*.

[16] Chowdhury N. H., Reaz M. B., Haque F. (2021). Performance analysis of Conventional machine learning algorithms for identification of chronic kidney disease in type 1 diabetes mellitus patients. *Diagnostics*.

[17] Allen A., Iqbal Z., Green-Saxena A. (2022). Prediction of diabetic kidney disease with machine learning algorithms, upon the initial diagnosis of type 2 diabetes mellitus. *BMJ Open Diabetes Research & Amp; Care*.

[18] Al-Rubeaan K., Siddiqui K., Alghonaim M., Youssef A. M., AlNaqeb D. (2018). The Saudi Diabetic Kidney Disease study (Saudi-DKD): clinical characteristics and biochemical parameters. *Annals of Saudi Medicine*.

[19] Senan E. M., Al-Adhaileh M. H., Alsaade F. W. (2021). Diagnosis of chronic kidney disease using Effective classification algorithms and recursive feature Elimination techniques. *Journal of Healthcare Engineering*.

[20] Sobrinho A., Queiroz A. C. M. D. S., Silva L. D. Da, Costa E. D. B., Pinheiro M. E., Perkusich A. (2020). Computer-aided diagnosis of chronic kidney disease in developing Countries: a comparative analysis of machine learning techniques. *IEEE Access*.

[21] Almansour N. A., Syed H. F., Khayat N. R. (2019). Neural network and support vector machine for the prediction of chronic kidney disease: a comparative study. *Computers in Biology and Medicine*.

[22] Khanam J. J., Foo S. Y. (2021). A comparison of machine learning algorithms for diabetes prediction. *ICT Express*.

[23] Rady E.-H. A., Anwar A. S. (2019). Prediction of kidney disease stages using data mining algorithms. *Informatics in Medicine Unlocked*.

[24] Sohail M., Ahmed H. M., Shabbir M., Noor K. (2020). Predicting chronic kidney disease by using classification algorithms in. *WE!*.

[25] Almasoud M., Ward T. E. (2019). Detection of chronic kidney disease using machine learning algorithms with least number of predictors. *International Journal of Advanced Computer Science and Applications*.

[26] Sarada J., Lakshmi N. V. M. Data analytics on chronic kidney disease data.

[27] Zeynu S., Professor A., Patil S. (2018). Survey on prediction of chronic kidney disease using data mining classification techniques and feature selection. *Shruti Patil*.

[28] Jian Y., Pasquier M., Sagahyroon A., Aloul F. (2021). A machine learning Approach to predicting diabetes complications. *Healthcare*.

[29] Tan K. R., Seng J. J. B., Kwan Y. H. (2021). Evaluation of machine learning methods developed for prediction of diabetes complications: a systematic review. *Journal of Diabetes Science and Technology*.

[30] Rodriguez-Romero V., Bergstrom R. F., Decker B. S., Lahu G., Vakilynejad M., Bies R. R. (2019). Prediction of nephropathy in type 2 diabetes: an analysis of the ACCORD trial applying machine learning techniques. *Clinical and Translational Science*.

[31] Pasadana I. A., Hartama D., Zarlis M. (2019). Chronic kidney disease prediction by using different decision tree techniques. *Journal of Physics: Conference Series*.

[32] Zhao J., Gu S., McDermaid A. (2019). Predicting outcomes of chronic kidney disease from EMR data based on Random Forest Regression. *Mathematical Biosciences*.

