# Supplementary material for: Comparison of Different Machine Learning Techniques to Predict Diabetic Kidney Disease
Source: J Healthc Eng. 2022 Apr 1;2022:7378307. doi: 10.1155/2022/7378307 (PMC8993553; doi:10.1155/2022/7378307)
Supplement: Supplementary Materials — Supplementary Table 1. Comparison of advantages and disadvantages of different classifiers used in our study. [file 7378307.f1.docx]

| **Classifiers** | **Advantages** | **disadvantages** |
| --- | --- | --- |
| **Decision Tree** | 1. It is easy for data preparation during pre-processing when using the decision tree 2. Normalization and scaling of data is not required   Decision tree is not affecting by the missing values in the data | 1. It is instable with changes in the data. Even a smaller change can result in larger variation in the in the structure of the decision tree. 2. It is more time consuming especially during training.   It relatively expensive |
| **J48** | 1. Any attribute can be chosen as the root of the decision tree. 2. Each value can be created as a branch. 3. Cases are divided into the branches and the process is repeated until all cases on the branches have the same classes. | 1. It creates many empty branches that do not contribute to classification but only complicate the trees 2. It produces insignificant branches that could decrease the usability of decision trees. 3. It over fits the noisy data with examples from training data |
| **REP Tree** | 1. It can be used for both contiguous and categorical values. 2. It simple to use with variables having complex relationship 3. The influence of incorrect or missing values is minimal | 1. It is unstable with even a small change in the input data. 2. It is complicated to analyze the large tree models |
| **Multilayer Perceptron** | 1. It is useful with even a very large input data. 2. Even a complex non-linear data can be applied. 3. It learns models in real-time | 1. The extent of influence of dependent variables on the independent variables is unknown 2. It requires tuning of number of hidden neurons, layers, and iterations 3. It is very expensive and training with less powerful CPUs could be time consuming |
| **IBK** | 1. It does not require a training step. 2. There is no need of any assumptions to be met to implement this algorithm 3. It quickly adapts and responds to the changes in the input data | 1. Performance decreases with large datasets. 2. High dimensionality also affects the performance as it can complicate the distance calculation process. 3. It is sensitive to missing and noisy data |
| **Random Tree/**  **Random Forest** | 1. It works with both continues and categorical values 2. Normalization of data is not required 3. It can automate the missing data. | 1. It requires high computational power as it creates large number of trees 2. Training time is very long as it has to combine a large number of trees to identify the class. |
| **Naïve Bayes** | 1. It works swiftly than many other algorithms 2. It is better suited for multi-class prediction models 3. Naive Bayes classifier will require less training data and perform better if the assumption of independence of features holds. | 1. It assumes all the predictors are independent. 2. It assigns zero probability to categorical variables that were not available in the training dataset 3. The probability outputs are less reliable |
| **AdaBoostM1** | 1. It is less predisposed to overfitting 2. It can solve multiclass problems 3. It can improve the accuracy of weak classifiers | 1. **Noisy data and outliers** can affect the classification |
| **Hoeffding Tree** | 1. It can be started on any preexisting decision tree 2. It has a very reliable performance and has a sound theoretical guarantee | 1. Hoeffding Tree has a slow initial learning problem and the performance needs to be boosted with an extension 2. Hoeffding Tree will not classify when a tie occurs in the dataset. |

**Supplementary Table 1.** Comparison of advantages and disadvantages of different classifiers used in our study
